# Population-Based Drug Resistance Surveillance of Multidrug-Resistant Tuberculosis in Taiwan, 2007-2014

**DOI:** 10.1371/journal.pone.0165222

**Published:** 2016-11-15

**Authors:** Pei-Hua Chuang, Mei-Hua Wu, Shin-Yuan Fan, Keng-Yu Lin, Ruwen Jou

**Affiliations:** 1 Tuberculosis Research Center, Centers for Disease Control, Taipei, Taiwan, R.O.C; 2 Institute of Microbiology and Immunology, National Yang-Ming University, Taipei, Taiwan, R.O.C; Institut de Genetique et Microbiologie, FRANCE

## Abstract

**Objective:**

To determine the extent of drug resistance in multidrug-resistant tuberculosis (MDR-TB) cases, we conducted a retrospective, population-based analysis using drug susceptibility testing (DST) results of MDR *Mycobacterium tuberculosis* complex isolates obtained from 2007–2014 in Taiwan.

**Methods:**

*M*. *tuberculosis* isolates collected from 1,331 MDR-TB cases were included in this survey. Treatment histories, age, sex, chest radiograph and bacteriological results of patients were analyzed. Standard DST was performed to assess resistance to the following drugs: isoniazid (INH), rifampicin (RIF), streptomycin (SM), ethambutol (EMB), amikacin (AM), kanamycin (KM), capreomycin (CAP), ofloxacin (OFX), moxifloxacin (MOX), levofloxacin (LVX), gatifloxacin (GAT), para-aminosalicylate (PAS), ethionamide (EA), and pyrazinamide (PZA). The Cochran-Armitage trend test was used for statistical analysis.

**Results:**

We observed a significant increasing trend in portion of new MDR-TB cases, from 59.5% to 80.2% (*p* < 0.0001), and significant decreasing trend of portion in the 15-44-year-old age group (*p* < 0.05). Of the MDR *M*. *tuberculosis* isolates tested, 6.2% were resistant to AM, 8.6% were resistant to KM, 4.6% were resistant to CAP, 19.5% were resistant to OFX, 17.1% were resistant to MOX, 16.0% were resistant to LVX, 5.8% were resistant to GAT, 9.5% were resistant to PAS, 28.5% were resistant to EA and 33.3% were resistant to PZA. Fifty (3.8%) extensively drug-resistant TB cases were identified. No significant differences were found in drug resistance frequencies between new and previously treated MDR cases. However, we observed significant decreases in the rates of AM resistance (*p* < 0.05), OFX resistance (*p* < 0.00001), PAS resistance (*p* < 0.00001), EA resistance (*p* < 0.05) and PZA resistance (*p* < 0.05). Moreover, younger age groups had higher rates of resistance to fluoroquinolones.

**Conclusion:**

A policy implemented in 2007 to restrict the prescription of fluoroquinolones was shown to be effective. Our survey revealed a decreasing trend of resistance to PZA, OFX and AM, which suggests the feasibility of adopting a short-course regimen and demonstrates the effectiveness of our management program for MDR-TB.

## Introduction

Tuberculosis (TB) is a major infectious disease worldwide. The World Health Organization (WHO) estimated that there are 9.6 million new TB cases and 1.5 million deaths annually [1]. In 2014, there were 11,326 new TB cases and 591 deaths were notified in Taiwan. The incidence rate was 48.4 cases per 100,000 populations. The TB incidence increased with age and 52.1% of new TB cases were in the age group of 65 years old or over. Besides, the male to female ratio was 2.3 [2–3]. Because drug resistance represents a growing threat to public health and TB control, surveillance to monitor the emergence and spread of drug resistance is a crucial component of the national strategy to combat drug resistance. Multidrug-resistant TB (MDR-TB), defined as TB caused by *Mycobacterium tuberculosis* that is resistant to at least isoniazid (INH) and rifampicin (RIF), is an increasing problem in global TB control. In 2014, there were an estimated 480,000 cases of MDR-TB, comprising 5% of all incident TB cases (3.3% of new cases and 20.0% of previously treated cases) [1, 4]. The WHO therefore addressed MDR-TB as a public health crisis in 2014 [1]. In Taiwan, MDR-TB accounted for approximately 1% of annual new TB cases and 6% of retreatment cases [2].

Treatment outcomes for patients with MDR-TB are worse than those for patients with INH- and RIF-susceptible TB. Additionally, it was estimated that 9.0% of MDR-TB patients had extensively drug-resistant TB (XDR-TB), defined as MDR *M*. *tuberculosis* resistant to at least one of the fluoroquinolones and one of the following injectable drugs: amikacin, capreomycin and kanamycin [1]. Globally, only 48% of MDR-TB patients in the 2011 cohort of diagnosed cases were successfully treated. Moreover, the treatment success rate for XDR-TB patients in the 2011 cohort was only 22% [5].

Systematic surveillance of drug-resistant TB can help to understand the overall burden of disease and can guide its diagnosis, treatment, infection control and public health management. An increasing number of countries have established national TB drug resistance surveillance systems. These systems adopt a standard methodology for DST. Nevertheless, establishing surveillance for drug resistance at the country level requires strict principles: (1) the specimens should be representative of the country; (2) the standard methods for DST should be internationally recommended and undertaken by quality-assured laboratories; and (3) patients should be clearly classified as new or previously treated cases. There is generally a higher prevalence of resistance in previously treated cases than in new cases [6].

Because universal DST was adopted by laboratory services of the National TB Program for every culture-positive TB case, a Surveillance of Drug-Resistant TB program was proposed in 2007 to establish population-based surveillance in Taiwan [7]. We report the first retrospective survey of drug resistance trends of MDR *M*. *tuberculosis* and the demographic characteristics of MDR-TB cases confirmed during 2007–2014.

## Materials and Methods

### Study population

In our TB control program, an MDR-TB case has to be bacteriologically confirmed by the National Reference Mycobacteriology Laboratory at the Taiwan Centers for Disease Control (TCDC). We analyzed the first isolate of all 1,331 MDR-TB confirmed cases from 2007 to 2014. Cases were categorized based on clinical information entered in the National TB Registry. Briefly, a new case was defined as one who had never been reported and recorded as an TB case in the network system, while a previously treated case had been previously recorded as an TB case and was re-identified as relapse after completing treatment of a previous episode. We obtained patient demographics, clinical information, acid-fast bacilli (AFB) sputum smear results and chest radiological data from the National TB Registry.

### Drug susceptibility testing

MDR *M*. *tuberculosis* isolates were subjected to DST using the proportion method with 7H10 medium (Becton, Dickinson and Company, Spark, MD, USA). Resistance was defined as 1% of the colonies growing in the presence of the following critical concentrations of drugs: isoniazid (INH) 0.2 μg/ml, rifampicin (RIF) 1 μg/ml, streptomycin (SM) 2 μg/ml, ethambutol (EMB) 5 μg/ml; and second-line drugs: amikacin (AM) 6 μg/ml, kanamycin (KM) 6 μg/ml, capreomycin (CAP) 10 μg/ml, ofloxacin (OFX) 2 μg/ml, moxifloxacin (MOX) 0.5 μg/ml, levofloxacin (LVX) 1.0 μg/ml, gatifloxacin (GAT) 1 μg/ml, para-aminosalicylate (PAS) 8 μg/ml and ethionamide (EA) 10 μg/ml [8–9]. Pyrazinamide (PZA) susceptibility testing was performed using a BACTEC^TM^ MGIT^TM^ 960 PZA Kit (Becton, Dickinson and Company, Spark, MD, USA) with a BACTEC^TM^ MGIT^TM^ 960 system (Becton, Dickinson and Company, Spark, MD, USA) as recommended by the manufacturer [8–9]. The critical concentration of PZA is 100 μg/ml. INH, RIF, EMB, SM, AM, KM, CAP, OFX, PAS, EA and PZA were tested in 2007–2014; MOX and GAT were tested in 2010–2014; and LVX was tested in 2012–2014. MDR-TB is defined as an *M*. *tuberculosis* isolate that is resistant to at least INH and RIF. Extensively drug-resistant TB (XDR-TB) is defined as an MDR *M*. *tuberculosis* isolate that is resistant to at least one fluoroquinolone and one injectable drug, while pre-XDR-TB is defined as an MDR *M*. *tuberculosis* isolate that is resistant to either fluoroquinolone (pre-XDR-fluo) or at least one of the injectable drugs (pre-XDR-inj).

### Statistical analysis

Demographic and DST data were entered into a Microsoft Excel (Microsoft, Redmond, WA, USA) spreadsheet and then transferred to the Data Analysis and Guiding System Cloud (Chinese Association of R Software Research and Applications, Taiwan). Analyses were conducted to determine the frequency of drug resistance in new or previously treated cases. Statistical analysis was performed using the Cochran-Armitage trend test for the comparison of proportions; *p*< 0.05 was considered statistically significant.

### Ethic statement

This study was approved by the Institutional Review Board of Centers for Disease Control, Ministry of Health and Welfare (TwCDC IRB No. 103114). The study only retrospectively reviewed the TB registry, written informed consent of the participants was waived.

## Results

### Population characteristics

From 2007–2014, we registered 1,331 MDR-TB patients, including 965 (72.5%) males and 366 (27.5%) females (Table 1). The male to female ratio was 2.64, which was slightly higher than that of the general TB population (2.3) [2]. Overall TB and MDR-TB cases had a higher percentage of males; however, the percentage of female MDR-TB cases was higher in new cases (*p* < 0.05) (Table 1). The ratio of male to female MDR-TB cases in the previously treated group was 3.3, which was higher than the ratio of 2.4 in new cases. Of the 1,331 cases, we identified 50 XDR-TB. The ratio of male to female XDR-TB cases was 2.8. Additionally, the mean age of MDR-TB cases was 53.5 years old (range: 12 to 97 years). There was a significant increasing trend in new cases and a decreasing trend in previously treated cases (*p* < 0.0001) (Table A in S1 File).

**Table 1:** Characteristics of MDR-TB cases in Taiwan, 2007–2014.

	Total cases (%)	New cases (%)	Previously treated cases (%)
	1,331 (100)	926 (69.6)	405 (30.4)
Sex			
Female	366 (27.5)	272 (29.4)	94 (23.2)
Male	965 (72.5)	654 (70.6)	311 (76.8)
Age (mean ± SD)	53.4 ± 18.0	52.8 ± 18.6	55.0 ± 16.3
Chest radiography			
Normal	23 (1.7)	19 (2.1)	4 (1.0)
Abnormal without cavitation	883 (66.3)	616 (66.5)	267 (65.9)
Abnormal with cavitation	412 (31.0)	283 (30.6)	129 (31.9)
Abnormal but not related to TB	11 (0.8)	8 (0.9)	3 (0.7)
Unknown	2 (0.2)	0 (0.0)	2 (0.5)
Pleural effusion			
Yes	65 (4.9)	58 (6.3)	7 (1.7)
No	1,266 (95.1)	867 (93.6)	398 (98.3)
Extrapulmonary TB	63 (4.7)	18 (1.9)	45 (11.1)
Pleural TB	11 (17.5)	10 (20.8)	1 (6.7)
TB of lymph nodes	11 (17.5)	10 (20.8)	1 (6.7)
Skeletal TB	9 (14.3)	3 (6.3)	6 (40.0)
Gastrointestinal TB	5 (7.9)	4 (8.3)	1 (6.7)
Tuberculous meningitis	3 (4.8)	2 (4.2)	1 (6.7)
Cutaneous and ocular TB	1 (1.6)	1 (2.1)	0 (0.0)
Urogenital TB	1 (1.6)	0 (0.0)	1 (6.7)
Other organ TB	22 (34.9)	18 (37.5)	4 (26.7)
Smear result			
Positive	787 (59.1)	549 (59.3)	238 (58.8)
Negative	530 (39.8)	369 (39.8)	161 (39.8)
Unknown	14 (1.1)	8 (0.9)	6 (1.5)

Analysis of the age distribution showed that cases above 65 years of age accounted for 27.4% (13.7%-37.1%) of the total MDR-TB cases, followed by 20.0% and 21.8% for the 55-64-year-old and 45-54-year-old age groups, respectively (Table 1). Furthermore, we confirmed six childhood MDR-TB cases. In addition, we observed a decreasing trend in the 15-44-year-old age group (*p* < 0.05)(Table B in S1 File). Of the 1,331 MDR-TB cases, 131, 322, 183, 159, 158, 133, 129 and 116 *M*. *tuberculosis* isolates were tested in 2007, 2008, 2009, 2010, 2011, 2012, 2013 and 2014, respectively. Excluding two cases with no X-ray radiograph results, 412 (31.0%) cases had cavitation (Table 1). In this study population, 1,295 (97.3%) cases had pulmonary TB, 63 (4.7%) had extrapulmonary TB and 48 (3.6%) had both. Specifically, 65 (4.9%) cases had accompanying pleural effusion (Table 1). Besides, the lymph nodes TB, pleural TB and skeletal TB were major types of extrapulmonary TB (Table 1).

### Bacteriological data

Of the 1,331 MDR-TB cases, 787 (59.8%) were AFB smear-positive (Table 1). The median smear-positive rates were 59.1% (48.1%-68.7%), 59.3% (49.1%-72.0%) and 58.8% (38.6%-67.3%) for overall, new and previously treated MDR-TB cases, respectively. We observed that the smear-positive rate from 2012 to 2014 showed a significant increasing trend in new cases and a significant decreasing trend in previously treated cases (Table A in S1 File).

### Drug resistance

The drug resistance patterns of 1,331 MDR *M*. *tuberculosis* isolates are summarized in Table 2. We found that 10.8% of MDR-TB cases were resistant to all 4 first-line drugs (INH, RIF, EMB and PZA) and SM (Table 2). The resistance rates for SM (42.2%, 95% CI 38.8–45.4) and EMB (42.2%, 95% CI 34.7–51.1) were slightly higher than that of PZA (33.3%, 95% CI 28.1–38.5). There were no significant differences among resistance rates of the four major drugs between new and previously treated cases. However, SM (*p* < 0.05) and EMB had higher resistance rates (44.3% and 43.8%, respectively) in new cases compared to previously treated cases (37.6% and 38.6%, respectively). Consequently, resistance rates for SM+EMB (*p* < 0.05) were significantly higher in new cases (Table 2).

**Table 2:** Drug resistance prevalence of MDR-TB cases in Taiwan, 2007–2014.

	Total cases (%)	New cases (%)	Previously treated cases (%)
	1,331	926	405
Additional resistance to 3 drugs*:			
Streptomycin (n = 1,328**)	561 (42.2)	409 (44.3)	152 (37.6)
Ethambutol (n = 1,328**)	561 (42.2)	405 (43.8)	156 (38.6)
Pyrazinamide (n = 1,323)	440 (33.3)	291 (31.7)	149 (36.8)
Streptomycin+Ethambutol	292 (22.0)	218 (23.6)	74 (18.3)
Streptomycin+Pyrazinamide	234 (17.7)	161 (17.5)	73 (18.0)
Ethambutol+Pyrazinamide	237 (17.9)	162 (17.6)	75 (18.5)
Streptomycin+Ethambutol+Pyrazinamide	143 (10.8)	103 (11.2)	40 (9.9)
Additional resistance to second-line drugs:			
Amikacin (n = 1,248)	77 (6.2)	54 (6.2)	23 (6.2)
Kanamycin (n = 1,331)	114 (8.6)	76 (8.2)	38 (9.4)
Capreomycin (n = 1,331)	61 (4.6)	42 (4.5)	19 (4.7)
Ofloxacin (n = 1,331)	260 (19.5)	170 (18.4)	90 (22.2)
Moxifloxacin (n = 724)	124 (17.1)	87 (15.9)	37 (21.0)
Levofloxacin (n = 344)	55 (16.0)	40 (14.5)	15 (21.7)
Gatifloxacin (n = 893)	52 (5.8)	30 (4.6)	22 (9.4)
Para-aminosalicylate (n = 1,331)	126 (9.5)	79 (8.5)	47 (11.6)
Ethionamide (n = 1,331)	380 (28.5)	258 (27.9)	122 (30.1)
MDR-TB***	981 (73.7)	697 (75.3)	284 (70.1)
Pre-XDR fluo***	227 (17.1)	146 (15.8)	81 (20.0)
Pre-XDR inj***	73 (5.5)	48 (5.2)	25 (6.2)
XDR-TB***	50 (3.8)	35 (3.8)	15 (3.7)

*Calculated based on the number of drugs tested.

**Excludes 3 cases without drug susceptibility testing results for streptomycin and ethambutol.

***MDR-TB: *Mycobacterium tuberculosis* isolates resistant to isoniazid and rifampicin and/or ethambutol and/or streptomycin; Pre-XDR fluo: MDR *M*. *tuberculosis* isolates resistant to any fluoroquinolone; Pre-XDR inj: MDR *M*. *tuberculosis* isolates resistant to any injectable drug; XDR-TB: extensively drug-resistant tuberculosis.

In contrast, new cases had lower resistance rates to second-line drugs than previously treated cases. There were significantly higher resistance rates to fluoroquinolones in previously treated cases (*p* < 0.05) compared to new cases, while this was not observed with injectable drugs. Accordingly, there were more pre-XDR cases with drug resistance to fluoroquinolones than cases with resistance to injectable drugs (Table 2). In this survey, we identified 50 (3.8%) XDR cases, 227 (17.1%) pre-XDR-fluo cases, and 73 (5.5%) pre-XDR-inj cases including 35 (47.9%) cases resistant to all 3 injectable drugs (Table 2). Of the 50 XDR-TB cases, 13 (26%) were female, and 37 (74%) were male. In addition, 12 (92.3%) female and 23 (62.2%) male cases were new XDR-TB cases.

Of the MDR isolates tested, 6.2% (95% confidence interval [CI] 3.0–9.4) were resistant to AM, 8.6% (95% CI 6.8–10.4) were resistant to KM, 4.6% (95% CI 3.4–5.8) were resistant to CAP, 19.5% (95% CI 12.1–31.6) were resistant to OFX, 17.1% (95% CI 14.3–19.4) were resistant to MOX, 16.0% (95% CI 13.7–18.3) were resistant to LVX, 5.8% (95% CI 4.6–7.0) were resistant to GAT, 9.5% (95% CI 5.2–13.8) were resistant to PAS, 28.5% (95% CI 23.6–33.4) were resistant to EA and 33.3% (95% CI 28.1–38.5) were resistant to PZA. We also observed significant decreasing trends of resistance to AM (*p* < 0.05), KM (*p* < 0.05), OFX (*p* < 0.0001), PAS (*p* < 0.0001) and PZA (*p* < 0.05) among total cases. Moreover, OFX and PAS resistance rates significantly decreased in new (*p* < 0.0001) and previously treated cases (*p* < 0.05)(Table B in S1 File). Moreover, resistance to fluoroquinolones was higher in younger age groups among new cases. We did not observe statistically significant differences among previously treated cases for any tested drugs. In the 15-24-year-old age group, PZA resistance (37.9%) was higher in new cases (n = 61) than in previously treated cases (n = 7), but resistance to PAS (14.3%) and EA (42.9%) was higher in previously treated cases (Table C in S1 File).

## Discussion

This population-based survey conducted from 2007 to 2014 in Taiwan has made the trend and extent of drug resistance of MDR-TB available for the first time. The quality of the DST is assured by participation in the External Quality Assessment of the World Health Organization (WHO) Supranational Reference Laboratory Network, the representativeness and accuracy of the survey can be assured. In Taiwan, *M*. *tuberculosis* resistance to INH is 9% and 18% in new and previously treated all TB cases, respectively, and resistance to RIF is 2% and 10% [2]. Approximately 1% of annual confirmed TB cases were MDR-TB [2]. Although the number of annual confirmed TB and MDR-TB cases is decreasing in Taiwan, the incidence rate remains 1% and 6–7% in new and previously treated TB cases, respectively [2]. The WHO estimated that 5% of global TB cases have MDR-TB, and 3.3% and 20% were found in new TB and previously treated TB cases, respectively [1]. However, this survey revealed a 3% increase in new MDR-TB cases over the year, which was comparable to the 3.6% in the United Kingdom and less than the 20.7% in Botswana [10].

The survey revealed that the incidence of female MDR-TB cases was higher in new cases (*p* < 0.05). The ratio of male to female MDR-TB cases in previously treated cases was higher than in new cases. This may be because females are the main caregivers in households or health care facilities [11, 12], where the chance of being a primary MDR-TB case is relatively high. The higher rate of male MDR-TB cases in the previously treated group suggests possible acquisition from previous treatments.

Based on the National TB Registry, 51.6%-53.1% of all annual TB cases are found in the elderly age group over 65 years old [2], whereas 27.9% (13.7%-37.1%) of MDR-TB cases are over 65 years old, and the majority (41.8%; 34.6%-48.1%) of MDR-TB cases in 2007–2014 are in the 45-64-year-old age group. The WHO reported that, in most countries, there is an age-dependent decrease in MDR-TB cases [4]. Following implementation of the enhanced MDR-TB management program in Taiwan in 2007, we observed decreasing trends of cases in the 15-44-year-old age group (*p* < 0.05). Chest radiographic data showed that 2.5% of MDR-TB cases did not have any TB-related lung abnormalities. Thus, it is not recommended to exclude TB cases by relying only on chest X-rays. Rapid bacteriological or molecular diagnostic tools are needed to confirm the final diagnosis. WHO recommended new tools for rapid detection and better treatment outcomes for MDR-TB through use of novel rapid diagnostic tests, the Xpert MTB/RIF assay (Cepheid, USA) and line-probe assays for detection of resistance to first and second line drugs (Hain LifeScience, Germany), and a shorter treatment regimen [13]. Moreover, we observed that AFB smear-positive rates in MDR-TB cases were higher than those of all TB cases (38%) [2]. Furthermore, there was a significant increasing trend of MDR-TB cases with positive AFB smears since 2012. This may be due to improvements in the quality of clinical TB laboratories, changes in the characteristics of cases or delays in diagnosis. Increasing numbers of new MDR cases observed in this survey indicate that smear-positive cases might cause recent transmission.

Globally, the estimated pooled PZA resistance in all TB and MDR-TB cases was 16.2% (95% CI 11.2–21.2) and 60.5% (95% CI 52.3–68.6), respectively [13]. In our previous survey, PZA resistance was 2% in 171 non-MDR-TB cases (unpublished data). This survey revealed that the rate of resistance to PZA was 33.3% (95% CI 28.1–38.5) in MDR-TB cases, which is relatively low compared to 48.8% (95% CI 30.1–67.5%) in the western Pacific, where Taiwan is located [14], and compared to Baltic countries, such as Estonia (71.4%) and Latvia (75.0%) [15]. However, in Estonia, rates of resistance to PZA showed no difference between new (71.4%) and previously treated cases (71.4%), and in Latvia, PZA resistance was higher in new cases (75.0%) than in previously treated cases (69.2%) [14]. However, there was a slightly higher rate of resistance in previously treated cases (36.8%) compared to new cases (31.7%) in Taiwan.

In 2013, surveillance of resistance to fluoroquinolones and PZA was initiated in five countries: Azerbaijan, Bangladesh, Belarus, Pakistan and South Africa. Preliminary results have shown that resistance to RIF is often associated with resistance to PZA. Moreover, resistance to OFX is generally lower than RIF resistance, except in Asian countries where fluoroquinolones are extensively used [4]. We report that the rate of resistance to OFX significantly decreased due to a policy implemented in 2007 to restrict the prescription of fluoroquinolones, which has been shown to be effective. In this survey, we observed a significant decrease in resistance to AM, KM, PAS and PZA, which is consistent with a previous study indicating that the direct observed therapy-short course policy can lower rates of resistance to anti-TB drugs [10]. These results may be useful to guide the implementation of short-course regimens for MDR-TB treatment [10].

A survey conducted by the Tuberculosis Research Committee in Japan revealed an age-dependent decreasing trend in resistance rates for all first-line anti-TB drugs in new cases [16]. Interestingly, our study showed that the same trend was observed in rates of resistance to fluoroquinolones, while there were age-dependent increases in resistance to three injectable drugs. However, the reasons underlying the differences among age groups and categories of MDR-TB cases remain unknown and merit further investigations.

By the end of 2014, at least one XDR-TB case was identified in 105 countries, and the WHO estimated that 9.7% of MDR-TB cases were XDR-TB [1]. Our previous report indicated that 10.2% of MDR-TB in 2005 was XDR-TB [7]. However, from 2007 to 2014, only 50 (3.8%) XDR-TB cases were confirmed, including 28 (56%) cases identified in 2007 and 2008. Following the establishment of an MDR-TB treatment consortium of the enhanced MDR management program in 2007, few XDR-TB cases (n = 2–5) were confirmed from 2009 to 2014. Data from the continuous surveillance of MDR-TB drug resistance over 8 years have informed and guided the response to our MDR-TB management program. Universal access to diagnosis and treatment of MDR-TB, resulting in decreased resistance to second-line drugs, indicates the considerable progress of the program in Taiwan [17]. However, it remains insufficient as new MDR-TB cases increased significantly, while the percentage (1%) of new MDR-TB cases in annual new notified TB cases remained unchanged. To further reduce the number of MDR-TB cases, our TB program has implemented priority actions, including preventing transmission, treating drug-susceptible TB cases, scaling up new rapid molecular tests for screening high-risk populations, and making quality treatment and care available for MDR-TB cases. In addition, our drug surveillance system is now expanding to cover more drugs, including group 3–5 drugs (cycloserine, clofazimine and linezolid) and repurposed drugs (oxyphenbutazone, meropenem, potassium clavulanate, amoxicillin, thioridazine, nitazoxanide, trimethoprim and sulfamethoxazole) [18–20] to determine the prevalence of resistance to and the epidemiological cut-off values of these drugs. The findings may be useful to guide clinical trials to develop better treatment regimens for MDR and XDR-TB.

## Supporting Information

S1 File Table A. Characteristics of MDR-TB cases in Taiwan, 2007–2014. Table B. Drug resistance prevalence of MDR-TB cases in Taiwan, 2007–2014. Table C. Drug resistance ratio of MDR-TB cases in Taiwan, by age groups*.
(DOCX)
