# Supplementary material for: Population-Based Drug Resistance Surveillance of Multidrug-Resistant Tuberculosis in Taiwan, 2007-2014
Source: PLoS One. 2016 Nov 15;11(11):e0165222. doi: 10.1371/journal.pone.0165222 (PMC5112772; doi:10.1371/journal.pone.0165222)
Supplement: S1 File — Table A. Characteristics of MDR-TB cases in Taiwan, 2007–2014. Table B. Drug resistance prevalence of MDR-TB cases in Taiwan, 2007–2014. Table C. Drug resistance ratio of MDR-TB cases in Taiwan, by age groups*. (DOCX) [file pone.0165222.s001.docx]

**Supplementary Information**

**Table A. Characteristics of MDR-TB cases in Taiwan, 2007-2014**

|  | | | **Total cases (%)** | | **New cases (%)** | **Previously treated cases (%)** |
| --- | --- | --- | --- | --- | --- | --- |
| **Year** | | | **1,331 (100)** | | **926 (69.6)** | **405 (30.4)** |
| ***P* value** | | |  | | **<0.0001** | **<0.0001** |
| **2007** | | | 131 (100) | | 78 (59.5) | 53 (40.5) |
| **2008** | | | 322 (100) | | 198 (61.5) | 124 (38.5) |
| **2009** | | | 183 (100) | | 122 (66.7) | 61 (33.3) |
| **2010** | | | 159 (100) | | 114 (71.7) | 45 (28.3) |
| **2011** | | | 158 (100) | | 113 (71.5) | 45 (28.5) |
| **2012** | | | 133 (100) | | 106 (79.7) | 27 (20.3) |
| **2013** | | | 129 (100) | | 102 (79.1) | 27 (20.9) |
| **2014** | | | 116 (100) | | 93 (80.2) | 23 (19.8) |
| **Sex**  ***P* value** | | | **0.8874** | | **0.2165** | **0.0622** |
| **Female** | | | **366 (27.5)** | | **272 (29.4)** | **94 (23.2)** |
| **2007** | | | 29 (22.1) | | 24 (30.8) | 5 (9.4) |
| **2008** | | | 84 (26.1) | | 57 (28.8) | 27 (21.8) |
| **2009** | | | 60 (32.8) | | 42 (34.4) | 18 (29.5) |
| **2010** | | | 51 (32.1) | | 38 (33.3) | 13 (28.9) |
| **2011** | | | 43 (27.2) | | 32 (28.3) | 11 (24.4) |
| **2012** | | | 34 (25.6) | | 30 (28.3) | 4 (14.8) |
| **2013** | | | 33 (25.6) | | 24 (23.5) | 9 (33.3) |
| **2014** | | | 32 (27.6) | | 25 (26.9) | 7 (30.4) |
| **Male** | | | **965 (72.5)** | | **654 (70.6)** | **311 (76.8)** |
| **2007** | | | 102 (77.9) | | 54 (69.2) | 48 (90.6) |
| **2008** | | | 238 (73.9) | | 141 (71.2) | 97 (78.2) |
| **2009** | | | 123 (67.2) | | 80 (65.6) | 43 (70.5) |
| **2010** | | | 108 (67.9) | | 76 (66.7) | 32 (71.1) |
| **2011** | | | 115 (72.8) | | 81 (71.7) | 34 (75.6) |
| **2012** | | | 99 (74.4) | | 76 (71.7) | 23 (85.2) |
| **2013** | | | 96 (74.4) | | 78 (76.5) | 18 (66.7) |
| **2014** | | | 84 (72.4) | | 68 (73.1) | 16 (69.6) |
| **Age** | | |  | |  |  |
| **0-14** | | | **6 (0.5)** | | **6 (0.6)** | **0 (0.0)** |
| **2007** | | | 2 (1.5) | | 2 (2.6) | 0 (0.0) |
| **2008** | | | 2 (0.6) | | 2 (1.0) | 0 (0.0) |
| **2009** | | | 0 (0.0) | | 0 (0.0) | 0 (0.0) |
| **2010** | | | 1 (0.6) | | 1 (0.9) | 0 (0.0) |
| **2011** | | | 1 (0.6) | | 1 (0.9) | 0 (0.0) |
| **2012** | | | 0 (0.0) | | 0 (0.0) | 0 (0.0) |
| **2013** | | | 0 (0.0) | | 0 (0.0) | 0 (0.0) |
| **2014** | | | 0 (0.0) | | 0 (0.0) | 0 (0.0) |
| **15-24** | | | **68 (5.1)** | | **61 (6.6)** | **7 (1.7)** |
| **2007** | | | 7 (5.3) | | 5 (6.4) | 2 (3.8) |
| **2008** | | | 16 (5.0) | | 14 (7.1) | 2 (1.6) |
| **2009** | | | 15 (8.2) | | 14 (11.5) | 1 (1.6) |
| **2010** | | | 7 (4.4) | | 6 (5.3) | 1 (2.2) |
| **2011** | | | 7 (4.4) | | 7 (6.2) | 0 (0.0) |
| **2012** | | | 7 (5.3) | | 7 (6.6) | 0 (0.0) |
| **2013** | | | 5 (3.9) | | 4 (3.9) | 1 (3.7) |
| **2014** | | | 4 (3.4) | | 4 (4.3) | 0 (0.0) |
| **25-34** | | | **153 (11.5)** | | **114 (12.3)** | **39 (9.6)** |
| **2007** | | | 17 (13.0) | | 11 (14.1) | 6 (11.3) |
| **2008** | | | 33 (10.2) | | 19 (9.6) | 14 (11.3) |
| **2009** | | | 24 (13.1) | | 19 (15.6) | 5 (8.2) |
| **2010** | | | 20 (12.6) | | 15 (13.2) | 5 (11.1) |
| **2011** | | | 15 (9.5) | | 12 (10.6) | 3 (6.7) |
| **2012** | | | 20 (15.0) | | 15 (14.2) | 5 (18.5) |
| **2013** | | | 17 (13.2) | | 17 (16.7) | 0 (0.0) |
| **2014** | | | 7 (6.0) | | 6 (6.5) | 1 (4.3) |
| **35-44** | | | **183 (13.7)** | | **112 (12.1)** | **71 (17.5)** |
| **2007** | | | 24 (18.3) | | 11 (14.1) | 13 (24.5) |
| **2008** | | | 55 (17.1) | | 35 (17.7) | 20 (16.1) |
| **2009** | | | 20 (10.9) | | 10 (8.2) | 10 (16.4) |
| **2010** | | | 19 (11.9) | | 12 (10.5) | 7 (15.6) |
| **2011** | | | 22 (13.9) | | 14 (12.4) | 8 (17.8) |
| **2012** | | | 20 (15.0) | | 16 (15.1) | 4 (14.8) |
| **2013** | | | 13 (10.1) | | 7 (6.9) | 6 (22.2) |
| **2014** | | | 10 (8.6) | | 7 (7.5) | 3 (13.0) |
| **45-54** | | | **290 (21.8)** | | **207 (22.4)** | **83 (20.5)** |
| **2007** | | | 42 (32.1) | | 27 (34.6) | 15 (28.3) |
| **2008** | | | 75 (23.3) | | 52 (26.3) | 23 (18.5) |
| **2009** | | | 40 (21.9) | | 32 (26.2) | 8 (13.1) |
| **2010** | | | 23 (14.5) | | 16 (14.0) | 7 (15.6) |
| **2011** | | | 35 (22.1) | | 20 (17.7) | 15 (33.3) |
| **2012** | | | 21 (15.8) | | 18 (17.0) | 3 (11.1) |
| **2013** | | | 33 (25.6) | | 25 (24.5) | 8 (29.6) |
| **2014** | | | 21 (18.1) | | 17 (18.3) | 4 (17.4) |
| **55-64** | | | **266 (20.0)** | | **183 (19.8)** | **83 (20.5)** |
| **2007** | | | 21 (16.0) | | 12 (15.4) | 9 (17.0) |
| **2008** | | | 64 (19.9) | | 35 (17.7) | 29 (23.4) |
| **2009** | | | 30 (16.4) | | 19 (15.6) | 11 (18.0) |
| **2010** | | | 32 (20.1) | | 21 (18.4) | 11 (24.4) |
| **2011** | | | 30 (19.0) | | 22 (19.5) | 8 (17.8) |
| **2012** | | | 30 (22.6) | | 25 (23.6) | 5 (18.5) |
| **2013** | | | 28 (21.7) | | 22 (21.6) | 6 (22.2) |
| **2014** | | | 31 (26.7) | | 27 (29.0) | 4 (17.4) |
| **>65** | | | **365 (27.4)** | | **243 (26.2)** | **122 (30.1)** |
| **2007** | | | 18 (13.7) | | 10 (12.8) | 8 (15.1) |
| **2008** | | | 77 (23.9) | | 41 (20.7) | 36 (19.0) |
| **2009** | | | 54 (29.5) | | 28 (23.0) | 26 (42.6) |
| **2010** | | | 57 (35.8) | | 43 (37.7) | 14 (31.1) |
| **2011** | | | 48 (30.4) | | 37 (32.7) | 11 (24.4) |
| **2012** | | | 35 (26.3) | | 25 (23.6) | 10 (37.0) |
| **2013** | | | 33 (25.6) | | 27 (26.5) | 6 (22.2) |
| **2014** | | | 43 (37.1) | | 32 (34.4) | 11 (47.8) |
| **Smear result** | | |  | |  |  |
| **Positive** | | | 787 (59.1) | | 549 (59.3) | 238 (58.8) |
| **2007** | | | 88 (68.2) | | 53 (68.8) | 35 (67.3) |
| **2008** | | | 211 (66.8) | | 125 (64.1) | 86 (70.5) |
| **2009** | | | 96 (52.7) | | 63 (51.6) | 33 (55.0) |
| **2010** | | | 76 (48.1) | | 59 (51.8) | 17 (38.6) |
| **2011** | | | 90 (57.3) | | 64 (56.6) | 26 (57.8) |
| **2012** | | | 68 (51.1) | | 52 (49.1) | 16 (59.3) |
| **2013** | | | 79 (62.2) | | 64 (64.0) | 14 (51.9) |
| **2014** | | | 79 (68.7) | | 67 (72.0) | 11 (50.0) |
| **Negative** | | | 530 (39.8) | | 369 (39.8) | 161 (39.8) |
| **Unknown** | | | 14 (1.1) | | 8 (0.9) | 6 (1.5) |
|  |  |  | |  |  |  |

**Table B. Drug resistance prevalence of MDR-TB cases in Taiwan, 2007-2014**

|  | **Total cases (%)** | | **New cases (%)** | **Previously treated cases (%)** |
| --- | --- | --- | --- | --- |
|  | **1,331** | | **926** | **405** |
| **Additional resistance to 3 drugs*:** |  | |  |  |
| **Streptomycin (n=1,328**)** | **561 (42.2)** | | **409 (44.3)** | **152 (37.6)** |
| **2007** | 61 (47.3) | | 39 (50.6) | 22 (42.3) |
| **2008** | 109 (33.7) | | 71 (35.7) | 38 (30.6) |
| **2009** | 78 (42.9) | | 56 (46.3) | 22 (36.1) |
| **2010** | 74 (46.5) | | 52 (45.6) | 22 (48.9) |
| **2011** | 76 (48.1) | | 54 (47.8) | 22 (48.9) |
| **2012** | 59 (44.4) | | 49 (46.2) | 10 (37.0) |
| **2013** | 54 (41.9) | | 47 (46.1) | 7 (25.9) |
| **2014** | 50 (43.1) | | 41 (44.1) | 9 (39.1) |
| ***P* value** | **0.1888** | | **0.3649** | **0.7028** |
| **Ethambutol (n=1,328**)** | **561 (42.2)** | | **405 (43.8)** | **156 (38.6)** |
| **2007** | 31 (24.2) | | 19 (24.7) | 12 (23.5) |
| **2008** | 102 (31.8) | | 62 (31.5) | 40 (32.3) |
| **2009** | 89 (49.2) | | 62 (51.7) | 27 (44.3) |
| **2010** | 71 (44.7) | | 53 (46.5) | 18 (40.0) |
| **2011** | 81 (51.3) | | 59 (52.2) | 22 (48.9) |
| **2012** | 58 (43.6) | | 47 (44.3) | 11 (40.7) |
| **2013** | 65 (50.4) | | 50 (49.0) | 15 (55.6) |
| **2014** | 64 (55.2) | | 53 (57.0) | 11 (47.8) |
| ***P* value** | **<0.0001** | | **<0.0001** | **0.0015** |
| **Pyrazinamide (n=1,323)** | **440 (33.3)** | | **291 (31.7)** | **149 (36.8)** |
| **2007** | 63 (48.1) | | 37 (47.4) | 26 (49.1) |
| **2008** | 120 (37.3) | | 68 (34.3) | 52 (41.9) |
| **2009** | 46 (25.1) | | 32 (26.2) | 14 (23.0) |
| **2010** | 48 (30.2) | | 30 (26.3) | 18 (40.0) |
| **2011** | 57 (36.1) | | 39 (34.5) | 18 (40.0) |
| **2012** | 34 (27.0) | | 28 (28.3) | 6 (22.2) |
| **2013** | 36 (27.9) | | 29 (28.4) | 7 (25.9) |
| **2014** | 36 (31.3) | | 28 (30.4) | 8 (34.8) |
| ***P* value** | **0.0036** | | **0.0630** | **0.0399** |
| **Streptomycin+Ethambutol** | **292 (22.0)** | | **218 (23.6)** | **74 (18.3)** |
| **2007** | 21 (67.7) | | 13 (68.4) | 8 (66.7) |
| **2008** | 45 (44.1) | | 28 (45.2) | 17 (42.5) |
| **2009** | 47 (52.8) | | 34 (54.8) | 13 (48.1) |
| **2010** | 42 (59.2) | | 33 (62.3) | 9 (50.0) |
| **2011** | 43 (53.1) | | 31 (52.5) | 12 (54.5) |
| **2012** | 28 (48.3) | | 21 (44.7) | 7 (63.6) |
| **2013** | 36 (55.4) | | 31 (62.0) | 5 (33.3) |
| **2014** | 30 (46.9) | | 27 (50.9) | 3 (27.3) |
| ***P* value** | **0.7117** | | **0.9475** | **0.3561** |
| **Streptomycin+Pyrazinamide** | **234 (17.7)** | | **161 (17.5)** | **73 (18.0)** |
| **2007** | 34 (54.8) | | 20 (54.1) | 14 (56.0) |
| **2008** | 51 (42.5) | | 30 (44.1) | 21 (40.4) |
| **2009** | 25 (55.6) | | 20 (64.5) | 5 (35.7) |
| **2010** | 30 (62.5) | | 21 (70.0) | 9 (50.0) |
| **2011** | 34 (59.6) | | 20 (51.3) | 14 (77.8) |
| **2012** | 14 (41.2) | | 12 (42.9) | 2 (33.3) |
| **2013** | 24 (66.7) | | 21 (72.4) | 3 (42.9) |
| **2014** | 22 (61.1) | | 17 (60.7) | 5 (62.5) |
| ***P* value** | **0.0613** | | **0.1741** | **0.3162** |
| **Ethambutol+Pyrazinamide** | **237 (17.9)** | | **162 (17.6)** | **75 (18.5)** |
| **2007** | 20 (32.3) | | 11 (29.7) | 9 (36.0) |
| **2008** | 52 (43.3) | | 27 (39.7) | 25 (48.1) |
| **2009** | 24 (53.3) | | 18 (58.1) | 6 (42.9) |
| **2010** | 29 (60.4) | | 19 (63.3) | 10 (55.6) |
| **2011** | 37 (64.9) | | 26 (66.7) | 11 (61.1) |
| **2012** | 17 (50.0) | | 14 (50.0) | 3 (50.0) |
| **2013** | 29 (80.6) | | 23 (79.3) | 6 (85.7) |
| **2014** | 29 (80.6) | | 24 (85.7) | 5 (62.5) |
| ***P* value** | **<0.0001** | | **<0.0001** | **0.0232** |
| **Streptomycin+Ethambutol+Pyrazinamide** | **143 (10.8)** | | **103 (11.2)** | **40 (9.9)** |
| **2007** | 12 (60.0) | | 6 (54.5) | 6 (66.7) |
| **2008** | 24 (46.2) | | 13 (48.1) | 11 (44.0) |
| **2009** | 18 (75.0) | | 15 (83.3) | 3 (50.0) |
| **2010** | 21 (72.4) | | 16 (84.2) | 5 (50.0) |
| **2011** | 22 (59.5) | | 14 (53.8) | 8 (72.7) |
| **2012** | 8 (47.1) | | 6 (42.9) | 2 (66.7) |
| **2013** | 20 (69.0) | | 17 (73.9) | 3 (50.0) |
| **2014** | 18 (62.1) | | 16 (66.7) | 2 (40.0) |
| ***P* value** | **0.3440** | | **0.4891** | **0.9184** |
| **Additional resistance to second-line drugs:** | |  |  |  |
| **Amikacin (n=1,248)** | **77 (6.2)** | | **54 (6.2)** | **23 (6.2)** |
| **2007** | 9 (12.8) | | 5 (16.7) | 4 (21.1) |
| **2008** | 16 (5.3) | | 11 (5.6) | 5 (4.1) |
| **2009** | 13 (6.8) | | 9 (7.4) | 4 (6.6) |
| **2010** | 10 (6.0) | | 6 (5.3) | 4 (8.9) |
| **2011** | 12 (8.1) | | 10 (8.8) | 2 (4.4) |
| **2012** | 5 (3.7) | | 5 (4.7) | 0 (0.0) |
| **2013** | 6 (4.6) | | 5 (4.9) | 1 (3.7) |
| **2014** | 6 (5.2) | | 3 (3.3) | 3 (4.7) |
| ***P* value** | **0.0436** | | **0.1229** | **0.0437** |
| **Kanamycin (n=1,331)** | **114 (8.6)** | | **76 (8.2)** | **38 (9.4)** |
| **2007** | 16 (11.6) | | 9 (11.5) | 7 (13.2) |
| **2008** | 29 (10.3) | | 17 (8.6) | 12 (9.7) |
| **2009** | 18 (11.1) | | 12 (9.8) | 6 (9.8) |
| **2010** | 14 (8.4) | | 9 (7.9) | 5 (11.1) |
| **2011** | 16 (10.6) | | 13 (11.5) | 3 (6.7) |
| **2012** | 6 (4.4) | | 6 (5.7) | 0 (0.0) |
| **2013** | 7 (5.3) | | 6 (5.9) | 1 (3.7) |
| **2014** | 8 (6.9) | | 4 (4.3) | 4 (5.4) |
| ***P* value** | **<0.0001** | | **0.0600** | **0.9232** |
| **Capreomycin (n=1,331)** | **61 (4.6)** | | **42 (4.5)** | **19 (4.7)** |
| **2007** | 9 (7.5) | | 4 (5.1) | 5 (9.4) |
| **2008** | 13 (5.0) | | 10 (5.1) | 3 (2.4) |
| **2009** | 9 (5.3) | | 5 (4.1) | 4 (6.6) |
| **2010** | 7 (4.2) | | 3 (2,6) | 4 (8.9) |
| **2011** | 11 (7.5) | | 9 (8.0) | 2 (4.4) |
| **2012** | 6 (4.4) | | 6 (5.7) | 0 (0.0) |
| **2013** | 4 (3.1) | | 4 (3.9) | 0 (0.0) |
| **2014** | 2 (1.7) | | 1 (1.1) | 1 (3.1) |
| ***P* value** | **0.1967** | | **0.4143** | **0.2612** |
| **Ofloxacin (n=1,331)** | **260 (19.5)** | | **170 (18.4)** | **90 (22.2)** |
| **2007** | 55 (44.2) | | 34 (43.6) | 21 (39.6) |
| **2008** | 78 (24.1) | | 47 (23.7) | 31 (25.0) |
| **2009** | 37 (21.6) | | 23 (18.9) | 14 (23.0) |
| **2010** | 15 (10.2) | | 11 (9.6) | 4 (8.9) |
| **2011** | 29 (18.0) | | 21 (18.6) | 8 (17.8) |
| **2012** | 19 (14.7) | | 16 (15.1) | 3 (11.1) |
| **2013** | 16 (12.2) | | 12 (11.8) | 4 (14.8) |
| **2014** | 11 (9.5) | | 6 (6.5) | 5 (12.4) |
| ***P* value** | **<0.0001** | | **<0.0001** | **0.0040** |
| **Moxifloxacin (n=724)** | **124 (17.1)** | | **87 (15.9)** | **37 (21.0)** |
| **2007** | ND*** | | ND | ND |
| **2008** | ND | | ND | ND |
| **2009** | ND | | ND | ND |
| **2010** | 21 (13.9) | | 13 (11.4) | 8 (17.8) |
| **2011** | 29 (18.1) | | 20 (17.7) | 9 (20.5) |
| **2012** | 20 (15.4) | | 17 (16.0) | 3 (11.1) |
| **2013** | 17 (13.0) | | 13 (12.7) | 4 (14.8) |
| **2014** | 11 (9.5) | | 6 (6.5) | 5 (13.2 |
| ***P* value** | **0.2169** | | **0.1949** | **0.9597** |
| **Levofloxacin (n=344)** | **55 (16.0)** | | **40 (14.5)** | **15 (21.7)** |
| **2007** | ND | | ND | ND |
| **2008** | ND | | ND | ND |
| **2009** | ND | | ND | ND |
| **2010** | ND | | ND | ND |
| **2011** | 2 (11.7) | | 2 (12.1) | 0 |
| **2012** | 8 (7.8) | | 7 (6.9) | 1 (9.1) |
| **2013** | 10 (8.6) | | 7 (5.4) | 3 (11.1) |
| **2014** | 10 (15.7) | | 5 (16.2) | 5 (21.7) |
| ***P* value** | **0.0664** | | **0.0125** | **0.2637** |
| **Gatifloxacin (n=893)** | **52 (5.8)** | | **30 (4.6)** | **22 (9.4)** |
| **2007** | ND | | ND | ND |
| **2008** | ND | | ND | ND |
| **2009** | ND | | ND | ND |
| **2010** | 4 (3.6) | | 3 (2.6) | 1 (2.2) |
| **2011** | 8 (5.0) | | 7 (6.2) | 1 (2.2) |
| **2012** | 5 (3.9) | | 3 (3.0) | 2 (7.4) |
| **2013** | 2 (1.5) | | 1 (1.0) | 1 (3.7) |
| **2014** | 3 (2.6) | | 0 (0.0) | 3 (1.6) |
| ***P* value** | **0.5057** | | **0.0421** | **0.0685** |
| **Para-aminosalicylate (n=1,331)** | **126 (9.5)** | | **79 (8.5)** | **47 (11.6)** |
| **2007** | 30 (23.8) | | 17 (21.8) | 13 (24.5) |
| **2008** | 37 (12.4) | | 19 (9.6) | 18 (14.4) |
| **2009** | 19 (11.1) | | 13 (10.7) | 6 (9.8) |
| **2010** | 11 (8.4) | | 7 (6.1) | 4 (8.9) |
| **2011** | 13 (8.7) | | 11 (9.7) | 2 (4.4) |
| **2012** | 5 (3.7) | | 5 (4.7) | 0 (0.0) |
| **2013** | 6 (4.6) | | 5 (4.9) | 1 (3.7) |
| **2014** | 5 (4.3) | | 2 (2.2) | 3 (4.7) |
| ***P* value** | **<0.0001** | | **<0.0001** | **0.0018** |
| **Ethionamide (n=1,331)** | **380 (28.5)** | | **258 (27.9)** | **122 (30.1)** |
| **2007** | 21 (15.0) | | 11 (14.1) | 10 (18.9) |
| **2008** | 78 (23.8) | | 45 (22.7) | 33 (26.6) |
| **2009** | 68 (36.8) | | 45 (36.9) | 23 (37.7) |
| **2010** | 58 (36.1) | | 39 (34.2) | 19 (42.2) |
| **2011** | 50 (31.7) | | 36 (31.9) | 14 (31.1) |
| **2012** | 42 (31.6) | | 34 (32.1) | 8 (29.6) |
| **2013** | 35 (26.7) | | 25 (24.5) | 10 (37.0) |
| **2014** | 28 (24.1) | | 23 (24.7) | 5 (27.1) |
| ***P* value** | **0.1798** | | **0.2711** | **0.2898** |
| *Calculated based on the number of drugs tested. **Excludes 3 cases without drug susceptibility testing results for streptomycin and ethambutol.  *** ND: not done. | | | | |

| **Table C. Drug resistance ratio of MDR-TB cases in Taiwan, by age groups*** | | | |
| --- | --- | --- | --- |
|  | **Total cases (%)** | **New cases (%)** | **Previously treated cases (%)** |
|  | **1,331** | **926** | **405** |
| **Amikacin** | **77 (6.2)** | **54 (6.2)** | **23 (6.2)** |
| **0-14** | 0 | 0 | 0 |
| **15-24** | 2 (2.9) | 1 (1.6) | 1 (14.3) |
| **25-34** | 4 (2.6) | 3 (2.6) | 1 (2.6) |
| **35-44** | 5 (2.7) | 0 | 5 (7.0) |
| **45-54** | 9 (3.1) | 9 (4.3) | 0 |
| **55-64** | 19 (7.1) | 10 (5.5) | 9 (10.8) |
| **>65** | 38 (10.4) | 31 (12.8) | 7 (5.7) |
| ***P* value** | **<0.0001** | **0.103** | **0.7255** |
| **Kanamycin** | **114 (8.6)** | **76 (8.2)** | **38 (9.4)** |
| **0-14** | 0 | 0 | 0 (0.0) |
| **15-24** | 5 (7.4) | 4 (6.6) | 1 (14.3) |
| **25-34** | 5 (3.3) | 3 (2.6) | 2 (5.1) |
| **35-44** | 14 (7.7) | 6 (5.4) | 8 (11.3) |
| **45-54** | 20 (6.9) | 15 (7.2) | 5 (6.0) |
| **55-64** | 26 (9.8) | 15 (8.2) | 11 (13.3) |
| **>65** | 44 (12.1) | 33 (13.6) | 11 (9.0) |
| ***P* value** | **0.0015** | **0.0007** | **0.6564** |
| **Capreomycin** | **61 (4.6)** | **42 (4.5)** | **19 (4.7)** |
| **0-14** | 0 | 0 | 0 |
| **15-24** | 2 (2.9) | 1 (1.6) | 1 (14.3) |
| **25-34** | 2 (1.3) | 1 (0.9) | 1 (2.5) |
| **35-44** | 6 (3.3) | 2 (1.8) | 4 (5.4) |
| **45-54** | 10 (3.4) | 8 (3.9) | 2 (2.3) |
| **55-64** | 9 (3.4) | 5 (2.7) | 4 (4.8) |
| **>65** | 32 (8.8) | 25 (10.3) | 7 (5.3) |
| ***P* value** | **<0.0001** | **<0.0001** | **0.8587** |
| **Ofloxacin** | **260 (19.5)** | **170 (18.4)** | **90 (22.2)** |
| **0-14** | 2 (33.3) | 2 (33.3) | 0 (0.0) |
| **15-24** | 16 (23.3) | 15 (24.6) | 1 (14.3) |
| **25-34** | 31 (20.3) | 25 (21.9) | 6 (15.4) |
| **35-44** | 39 (21.3) | 21 (18.8) | 18 (25.4) |
| **45-54** | 64 (22.1) | 46 (22.2) | 18 (21.7) |
| **55-64** | 52 (19.5) | 28 (15.3) | 24 (28.9) |
| **>65** | 56 (15.3) | 33 (13.6) | 23 (18.9) |
| ***P* value** | **0.0108** | **0.0015** | **0.9625** |
| **Moxifloxacin** | **124 (17.1)** | **87 (15.9)** | **37 (21.0)** |
| **0-14** | 0 | 0 | 0 |
| **15-24** | 10 (14.7) | 9 (14.8) | 1 (14.3) |
| **25-34** | 16 (10.5) | 15 (13.2) | 1 (2.6) |
| **35-44** | 10 (5.5) | 6 (5.4) | 4 (5.6) |
| **45-54** | 27 (9.3) | 20 (9.7) | 7 (8.4) |
| **55-64** | 28 (10.5) | 18 (9.8) | 10 (12.0) |
| **>65** | 33 (9.0) | 19 (7.8) | 14 (11.5) |
| ***P* value** | **0.19** | **0.0113** | **0.0836** |
| **Levofloxacin** | **55 (16.0)** | **40 (14.5)** | **15 (21.7)** |
| **0-14** | 0 | 0 | 0 |
| **15-24** | 4 (5.9) | 3 (4.9) | 1 (14.3) |
| **25-34** | 6 (3.9) | 6 (5.3) | 0 |
| **35-44** | 5 (2.7) | 4 (3.6) | 1 (1.4) |
| **45-54** | 16 (5.5) | 12 (5.8) | 4 (4.8) |
| **55-64** | 8 (3.0) | 6 (3.3) | 2 (2.4) |
| **>65** | 16 (4.4) | 9 (3.7) | 7 (5.7) |
| ***P* value** | **0.1246** | **0.0358** | **0.5444** |
| **Gatifloxacin** | **52 (5.8)** | **30 (4.6)** | **22 (9.4)** |
| **0-14** | 0 | 0 | 0 |
| **15-24** | 5 (7.4) | 5 (8.2) | 0 |
| **25-34** | 6 (3.9) | 6 (5.3) | 0 |
| **35-44** | 8 (4.4) | 2 (1.8) | 6 (8.5) |
| **45-54** | 9 (3.1) | 7 (3.4) | 2 (2.4) |
| **55-64** | 9 (3.4) | 4 (2.2) | 5 (6.0) |
| **>65** | 15 (4.1) | 6 (2.5) | 9 (7.4) |
| ***P* value** | **0.2937** | **0.021** | **0.3252** |
| **Para-aminosalicylate** | **126 (9.5)** | **79 (8.5)** | **47 (11.6)** |
| **0-14** | 0 | 0 | 0 |
| **15-24** | 5 (7.4) | 4 (6.6) | 1 (14.3) |
| **25-34** | 10 (6.5) | 8 (7.0) | 2 (5.1) |
| **35-44** | 26 (14.2) | 20 (17.9) | 6 (8.5) |
| **45-54** | 31 (10.7) | 18 (8.7) | 13 (15.7) |
| **55-64** | 27 (10.2) | 12 (6.6) | 15 (18.1) |
| **>65** | 27 (7.4) | 17 (7.0) | 10 (8.2) |
| ***P* value** | **0.6515** | **0.3197** | **0.8** |
| **Ethionamide** | **380 (28.5)** | **258 (27.9)** | **122 (30.1)** |
| **0-14** | 0 | 0 | 0 |
| **15-24** | 15 (22.1) | 12 (19.7) | 3 (42.9) |
| **25-34** | 50 (32.7) | 37 (32.5) | 13 (33.3) |
| **35-44** | 57 (31.1) | 34 (30.4) | 23 (32.4) |
| **45-54** | 73 (25.2) | 51 (24.6) | 22 (26.5) |
| **55-64** | 75 (28.2) | 48 (26.2) | 27 (32.5) |
| **>65** | 110 (30.1) | 76 (31.3) | 34 (27.9) |
| ***P* value** | **0.4526** | **0.2046** | **0.4112** |
| **Pyrazinamide** | **440 (33.3)** | **291 (31.7)** | **149 (36.8)** |
| **0-14** | 0 | 0 | 0 |
| **15-24** | 23 (33.8) | 22 (36.1) | 1 (14.3) |
| **25-34** | 47 (30.7) | 33 (28.9) | 14 (35.9) |
| **35-44** | 68 (37.2) | 41 (36.6) | 27 (38.0) |
| **45-54** | 101 (34.8) | 68 (32.9) | 33 (39.8) |
| **55-64** | 85 (32.0) | 53 (29.0) | 32 (38.6) |
| **>65** | 116 (31.8) | 74 (30.5) | 42 (34.4) |
| ***P* value** | **0.8642** | **0.706** | **0.986** |

*Calculated based on the number of each age group: 0-14 year age group n= 6, 15-24 year age group n= 68, 25-34 year age group n = 153, 35-44 year age group n= 183, 45-54 year age group n= 290, 55-64 year age group n= 266, above 65 year age group n= 365.
